# Providing and receiving support through a tailor-made mobile app: a qualitative study on experience of professionals and family caregivers to persons with dementia

**DOI:** 10.1186/s12877-024-05151-6

**Published:** 2024-06-25

**Authors:** Aber Sharon Kagwa, Åsa Dorell, Hanne Konradsen, Sofia Vikström, Zarina Nahar Kabir

**Affiliations:** 1 Department of Neurobiology, Care Sciences and Society, Division of Nursing, NVS, Karolinska Institutet Alfred Nobels Allé, 23 141 83 Huddinge, Stockholm, Sweden; 2 Department of Gastroenterology, Herlev and Gentofte Hospital, Borgmester Ib Juuls Vej 1, Herlev, Copenhagen, 2730 Denmark; 3 Faculty of Health, University of Copenhagen, Blegdamsvej 3B, Copenhagen, 2200 Denmark; 4 Department of Neurobiology, Care Sciences and Society, Division of Occupational Therapy, NVS, Karolinska Institutet Alfred Nobels Allé 23 141 83 Huddinge, Stockholm, Sweden

**Keywords:** Dementia, Dementia care, eHealth, Family caregiver, mHealth, Mobile app, Social care professional, Support

## Abstract

**Background:**

The ageing population and the rise of persons with dementia (PWD) living at home have increased the need for support by family caregivers (FC). Research suggests that most FCs are unprepared for the complex role of informal caregiving. The use of mobile applications (apps) provide a cost-effective and efficient opportunity for community-based social care professionals to provide tailor-made support to FCs. The literature indicates that there are limited mobile apps available to meet the needs of the FCs to PWD living at home. The aim of this study was to explore how social care professionals and FCs to PWD living at home experience providing and receiving support through a tailor-made mobile app named STAV.

**Methods:**

A qualitative descriptive design was applied. Data were collected through semi-structured interviews with 11 community-based social care professionals and 19 FCs of PWD living at home. The data were analyzed inductively using thematic analysis.

**Results:**

The social care professionals and the FCs’ experience of providing and receiving support through a mobile app was represented by the following themes: Accessibility to support – Bridging the gap, Engaging from a distance, and Limitations of the support.

**Conclusions:**

This study highlights the need for FCs to PWD to receive support that is tailor-made to their needs as caregivers. The findings from this study can help community-based social care providers plan and organize long-distance support for FCs to PWD living at home. The findings further support the use of a mobile app as a complement to traditional means of support for FCs to PWD which can facilitate their knowledge, awareness, and self-care management.

**Supplementary Information:**

The online version contains supplementary material available at 10.1186/s12877-024-05151-6.

## Background

Dementia is a chronic, and progressive condition that leads to disability and dependency among older people. The prevalence of persons with dementia (PWD) is increasing with population ageing. In Sweden, 154,000 people were living with dementia in 2019 with an estimation of 250,000 by 2050 [1].

A fundamental principle in Swedish eldercare is the ageing in place policy. The policy promotes the right for individuals to stay in their private homes as long as possible according to their own will and capacity [2]. Subsequently, The National Board and Welfare, the Social Act (SOSFS; 2001:453) states that municipalities are required to provide care for persons with chronic illness, older persons, and persons with functional variation living at home [3]. Much of the health- and social care required is provided by family caregivers (FCs) [4], defined as those who provide unpaid care [5]. Most of this, often prolonged informal care, in Sweden is provided by older spouses [4] and women (i.e., adult daughters) [6]. Currently, approximately 60% of PWD are repoted to live at home [7] often relying on FCs for care [8]. Research from Sweden reported that 24% of the PWD in Sweden live at home without any use of eldercare services (e.g., respite care, daycare) [9], and about 50% do not receive any type of home care services [8].

The benefits of ageing in place for a PWD include extending the possibility of independent living and staying somewhere familiar [10]. Taking on informal caregiving for a family member has also been shown to provide meaning and a sense of purpose for some FCs [11]. However, the progressive and complex nature of dementia may also provide challenges for FCs regarding e.g., dressing, feeding, and medication management [12]. Additional challenges for FCs include social isolation, financial strain, and behavioural changes in the PWD [13]. The complexity of the PWDs’ increasing care needs warrants different management strategies [12] where the FCs often act as coordinator of care for the PWD [14]. The challenging role of being a FC to a PWD can lead to a high level of caregiver stress [15]. A few of the main causes of caregiver stress are the behavioural and personality changes in the PWD such as social withdrawal [16], agitation, aggressiveness, physiological distress, and restlessness [17]. Another cause of caregiver stress is the constant need for vigilance needed to care for a PWD with dependent behaviours [16]. High levels of caregiver stress are a major barrier for FCs to manage their own self-care needs [15] which can lead to negative outcomes such as insomnia, emotional stress, or depressive symptoms [18]. Caregiver stress is also one of the key predictors of institutionalization of the PWD [13].

The Swedish Health and Social Welfare recommends that support to FCs should be based on multi-component support, such as respite, practical support, home care services, and psychosocial support, and be adapted to their individual and changing needs [19]. However, the most common support provided to FCs is indirect, i.e., the support that is aimed directly towards the care recipient (i.e., home care, rehabilitation, and senior daycare) to relieve the FC in their caregiving role. The other type of support is often directly provided to the FC through community-based social care professionals. Social care professionals mostly consist of social workers, nurses, and nurse assistants, who often work part-time, combining their work with other responsibilities (i.e., clinical work) in the municipalities [20]. Social care professionals play a key role in coordinating and developing support for FCs such as education, information on available services, support with social aid officers, and individual- and group support. Social care professionals are also responsible for the education of other professionals in the municipalities such as nurse assistants regarding FCs’ situation and how they can be supported. It is common that social care professionals lack a job description and are therefore often left to self-organize their work, which means the support provided by them varies across Sweden [20].

A recent systematic review synthesized existing literature regarding the needs of FCs to PWD living at home [21]. The authors identified the importance of (i) being supported, including social and formal support (i.e., community resources); (ii) receiving and accessing information tailored to the FCs’ individual needs; (iii) being trained and educated to manage the PWD; and (iv) finding a balance between care duties for the PWD and their own needs, particularly for those caregivers who lived together with the care recipient [21]. Similarly, a Swedish study reported that FCs needed individualized information at their own pace, as the dementia of the care recipient progresses [22]. Despite this, a recent study reported difficulties for FCs in finding credible information to support their needs in providing care [12]. Furthermore, most available information was either irrelevant or not applicable to their individual needs as caregivers [12]. Therefore, FCs rely highly on healthcare professionals (i.e., nurses) to provide them with evidence-based information. In the same study, the FCs also reported frustration over the fragmentation of support and information to problems they faced while caring for a PWD. This fragmentation required them to turn to various sources for adequate support [12]. Subsequently, most FCs are unprepared for all facets of the complex role of informal caregiving [23].

The increasing need for support of FCs to PWD has driven innovative solutions delivered by mobile health (mHealth) [24] to be developed. mHealth is a subset of eHealth defined as the delivery of healthcare services using mobile and wireless information and communication technologies (ICT) such as mobile phones and tablets [25]. Research has also shown that technological solutions including internet-based interventions for PWD, and their FCs facilitate ageing in place [26]. mHealth solutions are suggested to be more accessible than web-based applications [27] and have been found, in high-income countries, to increase the efficiency of healthcare providers and save them time. mHealth solutions also provide a platform for real-time communication between the healthcare provider and the healthcare recipient which facilitates a collaborative decision-making process [27]. Furthermore, mHealth solutions also have the potential to increase access to services at a low cost, as well as provide opportunities for personalized and demand-driven support with the potential of reaching isolated individuals such as people who live at a distance from care services [28].

Despite the identified benefits of mHealth solutions, a recent systematic review reported a lack of mHealth interventions targeting FCs to PWD living at home [29]. An evaluation of existing mobile applications (hereafter referred to as mobile apps) for FCs to PWD showed that most of them were aimed towards the management of the care recipients’ needs. Only a minor subset of the available mobile apps targeted the FCs’ personal needs such as social networking and stress management. Additionally, only a few of those mobile apps included features related to stress-managing activities such as breathing exercises and journaling [30]. Furthermore, there are limited studies that explore the experiences of both FCs and care professionals even though the involvement of all stakeholders is required to reach a sustainable implementation of eHealth solutions [31, 32]. Therefore, this study seeks to fill that gap by exploring the perspectives of both social care professionals and FCs on their use of an mHealth solution. The aim of this study was to explore how social care professionals and FCs to PWD living at home, experience providing and receiving support through a tailor-made mobile app named STAV (described below).

## Methods

A qualitative descriptive design was used, as we wanted to capture participants’ perspectives and evaluations [33].

### Setting and participants

This qualitative study was conducted in two municipalities in Sweden, Stockholm County, and Västerbotten County.

Participants: Eleven social care professionals and 19 FCs totalling 30 participants were included in the study.

The social care professionals were recruited through purposive sampling. They were eligible for participation in the study if they worked in a municipality in Sweden and provided support to FCs to PWD living at home. The social care professionals included in this study were all women who worked in municipalities or senior day care centres and consisted of registered nurses specialized in dementia care (known as Silvia nurses in Sweden), nurse assistants specialized in dementia care (known as Silvia sisters in Sweden), dementia coordinators and social workers. Prior to the study, the social care professionals received an information package that included information regarding the intervention (described below) and the intervention tool which was a mobile app [34]. The social care professionals also had an introduction meeting with members of the research team regarding the project through a face-to-face or online meeting. Each social care professional delivered support through the mobile app to 1–9 FCs to PWD living at home.

The FCs were recruited through networks of social care professionals, local healthcare centres as well as traditional media channels (i.e., websites of the municipalities) and social media channels of FCs networks and dementia-related associations. The FCs were eligible for participation if they were adults (18+), had cared for a PWD living at home for a minimum of 6 months, possessed a smartphone and/or tablet, could read and write Swedish, and had access to broadband/internet at their own expense.

Of the nineteen FCs who participated in the qualitative interviews, 16 were women, and three men. Thirteen of the FCs were spouses, two cohabitating partners, three adult children, and one was a close friend of the PWD. The mean age of the FCs was 72 years (ranging from 53 years to 85 years).

### The intervention

The 8-week long intervention was delivered by the social care professionals in the form of professional support. The support was tailored to the individual needs of the FCs and delivered through a tailor-made and interactive mobile app named STAV (STöd till AnhörigVårdare: Support to family caregivers) [34]. The mobile app STAV was developed following a focus group interview with community-based social care professionals specialised in dementia care and individual interviews with FCs to PWD living at home which generated insights and ideas from user perspectives [22]. A further study on the app reported benefits of receiving support through the app such as filling a gap, a way of offloading, access to relevant information in one place etc [35]. It also identified the need for a proper introduction to the app and the need of overcome technical barriers [35]. STAV was available on IOS and Android digital devices. The support was delivered through a chat feature that enabled communication with the FCs. Other features of STAV included mindfulness sessions, a digital diary, and a collection of web links with updated and relevant information regarding dementia and caregiving [34].

### Data collection

Data was collected after the completion of the 8-week intervention. Semi-structured interviews were conducted to explore the participants’ experiences of providing and receiving support through the intervention tool STAV. The interviews were conducted face-to-face at the FC’s residence (*n* = 7), at the social care professional’s workplace (*n* = 3), by telephone (*n* = 15), or online via video calls (*n* = 5). The telephone or online options were offered either for the convenience of the participants or to adhere to the restrictions during the COVID-19 pandemic. A semi-structured interview guide, one for FCs and one for social care professionals was used for data collection (see Supplementary Table 1, Additional file 1). Probing follow-up questions such as “Can you further describe…” and “Can you give an example of…” were used for clarification purposes. The interviews were conducted between October 2021 and August 2023 (Mode: 35 min, Minimum: 14:20 min and Maximum: 58:25 min). The interviews were conducted by two of the authors (ASK and ÅD), audio recorded and transcribed verbatim.

### Data analysis

**Fig. 1:**
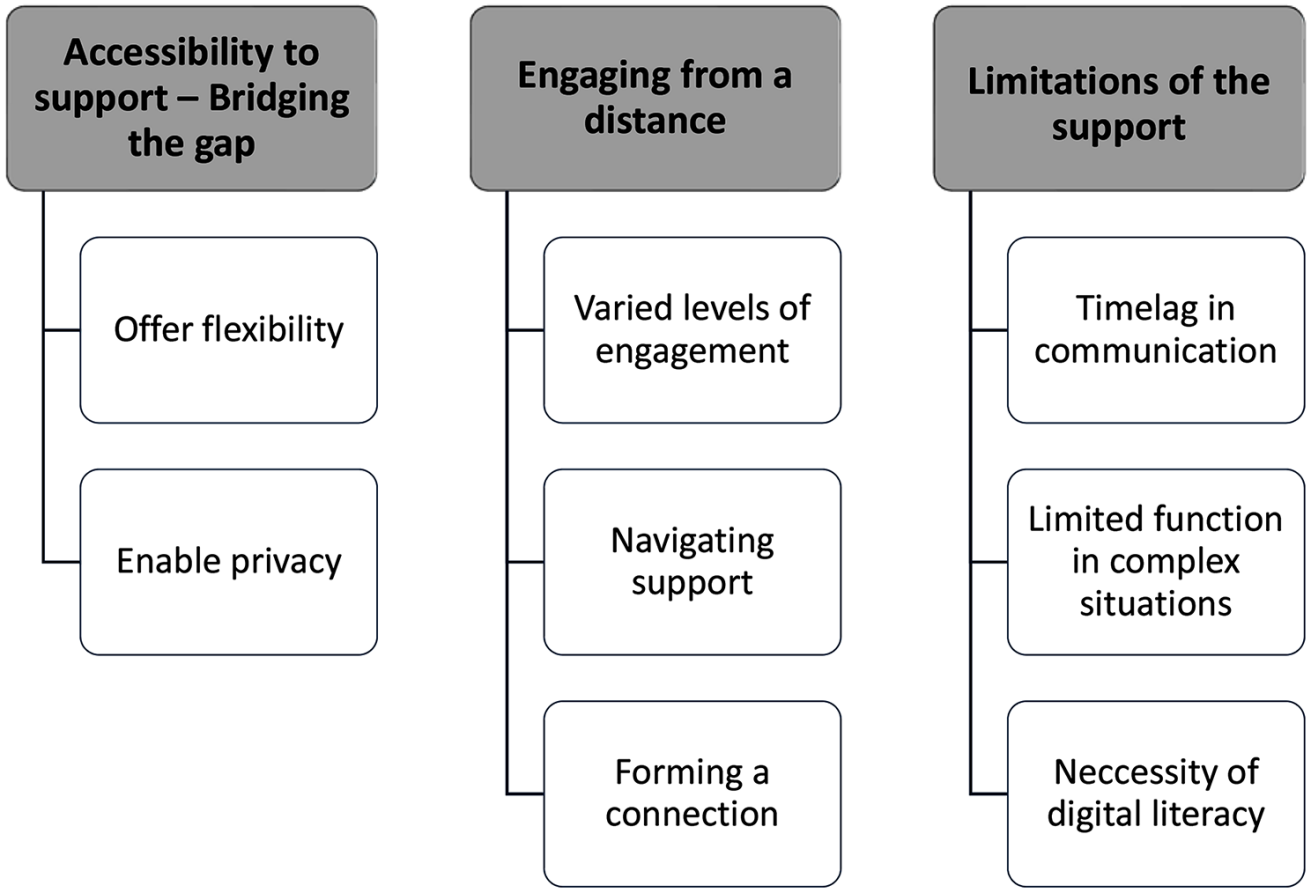
Overview of themes and subthemes

Thematic analysis with an inductive approach was used to give voice to the participants’ experiences of providing and receiving support through a mobile app. The analysis was guided by the six steps outlined by Braun and Clarke [36]; (i) Familiarization, (ii) Coding, (iii) Generating themes, (iv) Reviewing themes, (v) Defining and naming themes, and (vi) Producing the final report. It was a recursive process where the researchers moved back and forth in the six steps to address the study’s aim rather than following a linear process [37]. Interview data were managed using Microsoft Word and Microsoft Excel throughout the analysis. All the interviews were read by the first author (ASK). The other authors (ÅD, HK, SV, ZNK) read three interviews each. Initial codes were identified by all the authors independently. To ensure trustworthiness, the codes were then compared and combined by collating data relevant to each code. Similarities and differences were discussed between all the authors until a consensus was reached. Finally, overarching themes were identified, agreed upon, and named by all the authors together (Fig. 1). The final step was to produce the final report based on the themes. Quotes from the findings were also selected and interwoven in the results section to increase the trustworthiness of the study findings.

## Results

In this paper, we explored how social care professionals and FCs to PWD living at home experienced providing and receiving support through a mobile app. Three themes: (i) Accessibility to support – Bridging the gap; (ii) Engaging from a distance; (iii) Limitations of the support and eight subthemes emerged (Fig. 1).

### Accessibility to support – Bridging the gap

This theme relates to the benefits experienced in terms of accessibility to support by social care professionals and FCs while providing and receiving support through the mobile app STAV.

#### Offer flexibility

Flexibility was one of the main benefits expressed by both social care professionals and FCs of providing and receiving support through STAV respectively. The mode of communication enabled the FCs to communicate and ask questions to the social care professionals at their own pace, irrespective of time and place while caring for a dependent family member or friend. This was perceived as beneficial for the FCs by some of the social care professionals as well.

***Social care professional 1006:****… Some* [family caregivers] *find it convenient, I can ask my question in the chat any time of the day.*

Barriers such as office hours and the workload of the social care professionals were overcome by not having to maintain synchronous communication. Through the chat feature, the FCs could access support without waiting in telephone queues.

***FC1028****…with the app, you are not dependent on time, so to speak. If you call, there must be someone who answers and is available. But with the app, you can ask a few questions and get a response when they* [the social care professionals] *have time.*

Having STAV on a mobile device also enabled flexible use of the other support features of the app. The mindfulness exercises of various lengths were integrated into the daily lives of the FCs between care tasks and other activities. For example, one FC used the mindfulness feature while walking her dogs out in nature, while another FC used the diary feature at various places as she explained:

***FC1003:****…because it* [the diary] *was in my phone, I could write when I was sitting in the bus, or in a waiting room at the doctor’s, and then one can conclude and continue later.*

Flexibility was also appreciated by the social care professionals who expressed that the mode of communication enabled them to respond to questions and provide support at their own pace. STAV also enabled social care professionals to search for adequate answers or ask colleagues for advice. In contrast to providing support through the telephone which often demanded an immediate response support via the app resulted in more thought out and evidence-based support. The time for reflection before providing an answer also gave some of the social care professionals a sense of well-being. One social care professional expressed a reduced level of stress while providing support through STAV.

***Social care professional 1008:****It doesn’t become the same pressure and the same stress as me having to sit on the phone and provide that time.*

Furthermore, the social care professional also stated that she could multi-task and provide support through STAV while caring for her clients with dementia at the daycare centre.

#### Enable privacy

A benefit of STAV expressed by the participants was that it provided the FCs with privacy to communicate while caring for a PWD who exhibited dependent and suspicious behaviours. Having STAV on their personal devices enabled the FCs to communicate with the social care professionals about sensitive issues including concerns regarding the PWD without them accessing it.

***FC1003:****… I live here with my husband [the person with dementia], and I think it’s nice to be able to have it on my phone so I can write* [in STAV] *about him*, *and he cannot see it.*

The possibility for privacy was perceived by one social care professional as liberating for one FC.

***Social care professional 1002:****… It was so wonderful because you don’t have to have the husband* [the person with dementia] *go and read somewhere, he still can’t access that tablet so (laughter)…so it was like, she* [the family caregiver] *thought it was very liberating.*

Privacy was also discussed in terms of confidentiality by a couple of the social care professionals, as one social care professional explained:

***Social care professional 1009:****And I like that it is confidential. You can be completely sure of the confidentiality, and I like that, it is very important to me in my support to family caregivers.*

The privacy enabled by the mobile app STAV facilitated communication between the participants regarding sensitive subjects.

### Engaging from a distance

This theme relates to the level of engagement of the social care professionals and the FCs while providing and receiving support through the mobile app STAV. Engaging through the mobile app enabled the social care professionals to navigate support to the FCs and for the participants to form a connection. Engagement with the other app features also offered the FCs support, a platform reflection, and a source of inner calm.

#### Varied levels of engagement

The levels of engagement with STAV varied among the participants. Some of the social care professionals expressed that the FCs were less active than they had expected. In some cases, the participants didn’t know each other prior to the intervention. In those cases, it was mostly the social care professionals who initiated the conversations in the chat. Some FCs with low engagement with the app expressed that they cared for a relatively independent family member in the early stage of dementia. A few FCs also expressed that they had adjusted to their situation as caregivers.

The social care professionals took different approaches in the chat. Some waited for the FCs to initiate conversations while others were more proactive. They initiated conversations with the FCs on a regular basis using different approaches such as expressing interest in their situations or sending self-care reminders to maintain contact.

***Social care professional 1002:****…you probably started to poke around a bit, yes, but just like you do with other people when you’re trying to create a bond. You kind of have to show a little curiosity.*

The social care professionals also engaged by organizing their availability, ranging from a few times a week, creating specific time slots during the day, to daily contacts. Most social care professionals conveyed their availability to the FCs prior to the start of the intervention.

***Social care professional 1011:****We did say Tuesdays and Thursdays and sometimes the time changed a little, sometimes you could check it* [the chat] *in the morning, but then something came up in the afternoon so it was a bit like that.*

Some social care professionals took a more spontaneous approach or adjusted their level of activity by responding in the chat to meet the current needs of the FCs. Engagement from the social care professionals was appreciated by the FCs because they felt that they received timely and demand-driven support. Some social care professionals also showed a higher level of engagement in the intervention by reflecting on the quality of the other support features of STAV. A few social care professionals even discussed or recommended the other features (i.e., the mindfulness feature and the web links) to the FCs for additional support.

***Social care professional 2004:****…and these relaxation exercises* [mindfulness], *I have also recommended it to people* [family caregivers].

Lower engagement with the other support features in STAV was mostly based on the FCs’ personal situation or characteristics. Some FCs were already familiar with the information provided in the web links. Other FCs preferred to use pen and paper while writing in their diary. Some felt like the mindfulness feature was not suitable for them or achieved relaxation through other activities.

***FC1037:****… I sit by the sewing machine and there is my mindfulness. There I can relax.*

#### Engagement with technology

One FC perceived the app STAV as a source of stability that helped her to stay afloat in her daily life as a caregiver. The FC further expressed that it had taken her a while to realize that she could use STAV for her personal needs instead of solely focusing on the needs of the PWD whom she cared for.

***FC1042:****… this* [STAV] *functions as a… type of lifebuoy or….as an anchor in everyday life so I know this function is here.*

The diary feature enabled some FCs to vent and relieve stressful events regarding their role as caregivers. The diary was also used as a tool for reflection, where the FCs could look back on prior events such as conflicts that occurred while coordinating care with the healthcare system. Furthermore, the diary was also used to recall prior strategies used to deflect strenuous situations with the PWD and to track potential deterioration in the condition.

The mindfulness feature was also used for relaxation. Some FCs expressed feelings of inner calm while listening to or watching pictures of nature provided in the exercises.

***FC1031:****… there were very beautiful pictures, so it was very nice to look at them too.*

One FC expressed that she chose the thematic exercises (i.e., on the themes of relaxation and caring) depending on her current mood, and the level of anxiety she had in her body.

#### Navigating support

Through the chat feature the FCs received both practical and emotional support from the social care professionals. The practical support mostly concerned issues related to the PWD as discussions regarding different support services and aids (e.g., incontinence protection and walking aids).

***FC1028:****Yes, there were a few questions about my wife who is at the daycare centre…and it was mostly issues that concern her…yes, I got the response I wanted so it was ok.*

The social care professionals supported the FCs by guiding their conversations with social aid officers or other healthcare professionals.

***Social care professional 1007:****After all, I help to mediate contact…with the social aid officer and, and so on, so that was quite constructive to be able to use the app…or the chat in that way with him* [the family caregiver].

The social care professionals also facilitated the navigation to different support services by sending relevant web links and information such as future power of attorney documents in the chat.

#### Forming a connection

For most of the FCs, the chat feature in STAV provided a lower threshold to contact the social care professional compared to calling by telephone. In the chat, they felt comfortable asking questions, even concerning minor issues. Therefore, the chat as a support feature increased accessibility to the social care professional providing demand-driven support that they didn’t receive through traditional channels such as support groups or the telephone.

***FC1005:****…If I hadn’t had this app, then maybe we wouldn’t have had that contact and that conversation because I don’t call her* [the social care professional] *…. Then it has to be something special for me to call her* [the social care professional] *and ask her about.*

Receiving support through the chat enabled the FCs to easily connect with the social care professionals. The intervention helped establish an initial contact, for those FCs who didn’t have any prior contact with a social care professional. Other FCs stated that they appreciated the consistency of having one contact person, unlike when different staff members would respond when they called the daycare centre.

***FC1026:****… it makes it easier to still have some kind of continuity in the contact.*

The FCs expressed the importance of forming a human connection, especially during the COVID-19 pandemic. The chat feature was thus used to share both joyous and sad moments with the social care professionals.

Some social care professionals mentioned that the FCs used the chat feature to receive emotional support from them. Likewise, the chat was used by a few FCs to verify perceptions of changing behaviours in the PWD with the social care professionals. Some FCs used the chat as a sounding board, venting their frustrations to the social care professionals.

***Social care professional 1005:****Great! And just to get this* [app], *or for the family caregiver to be able to write this information down and vent…. worry and such, just like after a situation like this, it….is very valuable.*

Some FCs stated that their level of stress had reduced while receiving a timely response to their questions from the social care professionals which improved their sense of well-being.

***FC1003:****It reduces stress if you are worried about something, you can quickly get a response through a chat, for example, instead of calling and then it is busy, and then no one calls you back…*.

The social care professional also expressed an increased level of closeness to the FCs while using STAV. The chat feature enabled the professionals to gain a deeper understanding of their situation.

***Social care professional 1002:****… You got to know them* [the family caregivers] *at a deeper level this way.*

Hence, the chat was identified as a great complement of communication to traditional methods while providing brief information, and keeping regular contact with those FCs who needed it or those who preferred not to meet face-to-face. Using the chat as a complement to other means of support made the social care professionals work more efficiently. Some professionals reported that even though the introduction of STAV created an additional workload, it did reduce time spent on face-to-face meetings and telephone calls with the FCs.

***Social care professional 1001:****Yes, there will always be an added workload that you have to do or one more work task that has to be done. But at the same time, I believe […] that you can avoid having so many conversations. I think… it can complement other conversations and the app, it can help.*

Providing support through STAV was especially efficient while supporting FCs who needed daily or regular contact because it was easier to communicate through chat rather than sending emails each time.

### Limitations of the support

This theme relates to the challenges faced by social care professionals and FCs while providing and receiving support through the mobile app STAV which resulted in limitations of the support.

#### Timelag in communication

Most of the social care professionals and FCs found the usability of STAV as simple in terms of navigation and design which facilitated the use of its support features.

***FC1043:****No, the app was great, well, I think it was very easy to use and understand these sections* [app features]. *I think it was a great app, accessing it was no problem.*

However, some participants experienced challenges in communication while receiving and providing support through the chat. A challenge for some social care professionals was not knowing if the FCs had received their messages in the chat, especially when it took them a while to respond. A social care professional explained:

***Social care professional 1010***: *It’s a bit like when you have a lecture online you don’t really know what the recipient thinks if they have received it. It becomes a bit like this that you just wonder: “-Hello, are you there?” It was a bit like that.*

One FC expressed dissatisfaction over the delayed response from the social care professional. A social care professional also found it challenging to provide support through an asynchronous chat because the need of the FCs could be over by the time she responded to their questions.

### Limited function in complex situations

The participants especially expressed concerns in terms of miscommunication that sometimes occurred, as one FCs explained:

***FC1035:****…Then she* [the Silvia sister] *gave me advice and then I understood that I had not written properly because her answer did not correlate with what I had written to her. And then I felt that no, it went wrong, and it wasn’t her* [the Silvia sister’s] *fault.*

Some social care professionals also found it challenging to discuss complex issues through the chat. Therefore, the social care professionals adjusted their communication channels depending on the level of information they needed to provide. When the social care professionals needed to provide in-depth information or support regarding e.g., long-term care options they reverted to more traditional information channels such as emails or telephone calls.

***Social care professional 1001:****If there were some more complicated questions, I always called instead of writing, I called and took a conversation then.*

Non-verbal communication and not meeting face-to-face were restricting for some participants. One FC felt that they could address only one issue at a time through the chat. Other FCs expressed difficulties discussing complex and sensitive matters concerning their situation while caring for a PWD. The social care professionals also noticed that some FCs had a hard time opening up to share their concerns through the chat.

Some FCs expressed that they didn’t experience any benefit in terms of well-being in their daily lives. A FC expressed the need for more human contact than what the chat could offer. The mobile app also didn’t reduce their feelings of loneliness and isolation, as one FC explained:

***FC1005:****The isolation doesn’t get any less just because you have an app to communicate on, you’re just as isolated and alone anyway.*

A few of the social care professionals didn’t experience any benefit from using STAV in their daily work. This was mostly because they were already accessible to the FCs through other communication channels such as support groups.

#### Necessity of digital literacy

Both the social care professionals and the FCs expressed experiencing challenges for the FCs with the technicalities of using the app STAV, which they mostly related to age and a lack of experience. The lack of experience caused some FCs to disengage from the support features in STAV. Some social care professionals and FCs therefore stated that this mode of communication was more suitable for those who were digitally literate or younger.

***FC1034:****And it’s probably the fact that I’m still the generation that is, who doesn’t have apps in the first place.*

***Social care professional 2004:****I think it* [STAV] *suits a bit younger target group […] good for those who are used to using technology. So, it can be young people* [family caregivers] *such as those under 65.*

One social care professional also addressed the need for social care professionals to be digitally literate to provide adequate support through a mobile app.

***Social care professional 1009:****… but then there is the question whether…the person who works with family support is app friendly, you don’t know that.*

The lack of technical experience which caused some FCs to disengage with STAV resulted in disappointment. However, some participants felt that those FCs who experienced difficulties using the STAV had the possibility to learn and improve.

## Discussion

This study aimed to explore how social care professionals and FCs to PWD living at home experience providing and receiving support through a mobile app. This current study presents new insights into the benefits and challenges of providing and receiving support through a mobile app from two important user perspectives.

In this study *accessibility to support* was identified as one of the main benefits of providing and receiving support through a mobile app. Recent research indicates that the benefit of mHealth technologies is its mobility which doesn’t require the user to be at any specific place and removes distance-related constraints between the care provider and the care recipient [38]. The mobile app *offered flexibility* which enabled the social care professionals and the FCs to provide and receive support at their own pace, irrespective of time and place at their convenience. These results are in line with a previous Swedish study [35] that found the possibility for FCs to PWD living at home to have direct contact with healthcare professionals at a time and place that was convenient for them provided a sense of security for the FCs. The mobility of the mobile app also *enabled privacy* and confidentiality. Through the mobile app, the social care professionals and the FCs could communicate about sensitive subjects even when caring for a PWD with functional dependency and behavioural challenges (i.e., exhibiting suspicious behaviours) which further increased *accessibility to support.*

In our study, the social care professionals’ office hours or workload did not become a barrier to providing support, and neither were long telephone queues for FCs in reaching out to the professionals. Previous research reports that FCs to PWD can feel guilty about taking up the service staff’s time when talking about their personal needs [36]. The findings of this current study suggest that the flexibility of the mobile app that could be used irrespective of time and place mitigated these feelings for the FCs. The chat feature of the mobile app in this study also provided a lower threshold to support than traditional means of communication such as the telephone and participation in support groups. The chat feature provided a platform for the FCs to contact the social care professional even with minor issues which enabled the social care professionals to meet the FC’s individual needs. These findings are in line with previous research that has identified the potential for individualization and a low threshold of access, as some of the benefits of eHealth interventions, which alongside their low costs could facilitate widespread implementation [39]. Some social care professionals who showed higher levels of engagement regulated their availability to meet the individual needs of the FCs. The lack of previous contact between some of the social care professionals and the FCs might have influenced the *varied levels of engagement* in the chat feature. The varied engagement by the social care professionals could also be attributed to the fact that most social care professionals in Sweden are left to self-organize their work due to a lack of standardized job descriptions [20].

Through the chat feature the social care professionals and the FCs could *engage from a distance*, providing and receiving support. The chat feature enabled demand-driven and direct contact with social care professionals for the FCs to PWD. Through the chat, the social care professionals *navigated support* by providing the FCs with tailor-made information that increased the FCs’ awareness regarding available support services for dementia-related support, and self-care advice. Another benefit of mHealth technologies is that they provide the user with more time and flexibility to absorb information [25]. By improving access to information, mHealth technology has the potential to reduce information-related medical errors by providing the healthcare professional with the correct information when needed [40]. Similar benefits were also identified with the mobile app in this current study which enabled social care professionals to search for correct information and/or ask their colleagues before responding to the FCs in the chat feature which enabled them to provide adequate and evidence-based support. A recent systematic review identified that mHealth interventions can help FCs connect with their care team [41]. The FCs in this study expressed appreciation for having one contact person to *form a connection* with through the mobile app which created a sense of continuity. A study exploring factors related to self-care behaviours among FCs to PWD also identified the importance of having one key contact person to provide continuity of care and support as an important factor in meeting the needs of FCs [42].

The FCs engaged by using the mindfulness feature of the mobile app with a variety of exercises which was easily incorporated into their daily lives in between care tasks and other duties and provided a source of calm and self-focus, findings which are supported by a previous Swedish study [35]. These findings are important because previous research shows that FCs to PWD often feel unable to prioritize their own needs due to a lack of time and constant need for care provision. The time in between caring tasks for FCs to PWD is often used to sort out other necessities such as finances and household chores [42]. This is in line with findings from previous research [43] regarding the experience of caregivers to persons with cognitive impairment of using mindfulness therapy in a mobile app. The study found that flexibility and convenience were important for the usability and acceptability of the mobile app which could be incorporated according to the caregivers’ moods, schedules, and time preferences [43]. The findings in this study are valuable because research has shown that it is important for FCs to take a break by performing activities independent of the PWD to shift focus from being a caregiver, through rest and respite care [15]. Furthermore, mobile based mindfulness interventions have been shown to increase the well-being of FCs to PWD [44] and provide them with a foundation for inner calm [35]. The FCs also engaged by using the other support features of the mobile app such as the web links with useful information about dementia and caregiving also provided increased knowledge and awareness for the FCs which helped them in their daily caregiving tasks. These findings are aligned with previous research [45] which identified that eHealth solutions have the potential to increase access to health and social care which can support behaviour changes in individuals and support the performance of self-care activities.

The lack of technical experience and/or capacity to handle the app was one of the reasons some FCs disengaged. Research has shown that socio-demographic factors such as age affect digital literacy [46]. Therefore, the challenges faced by some of the FCs regarding digital literacy could be attributed to the fact that most of the FCs in this study were spouses and older persons themselves [4]. Other reasons for lower levels of engagement among the FCs included caring for a relatively independent PWD thus not yet requiring much support having adjusted to their situation as caregivers, and/or personal preferences (i.e., preferring pen and paper while writing a diary).

Another challenge addressed by the participants while providing and receiving support through a mobile app was the *time lag in communication.* Even though the overcoming of geographic constraints is identified as a benefit of mHealth technologies [40] it could also contribute to some of the challenges of delayed and/or misunderstanding in [43] communication through the mobile app. Some participants found it difficult to communicate through an asynchronous chat, while others expressed challenges with not knowing if the recipient had received their messages. The miscommunications that occurred between some of the social care professionals and the FCs could be related to *the necessity of digital literacy*. Some of the participants also found that the mobile app provided *limited function in complex situations s*uch as while discussing long-term care options through the chat feature. These challenges created *limitations of the support* and were often addressed by reverting to traditional means of communication such as telephone calls, emails, and face-to-face meetings. Thus, it may be necessary to recognize that the mobile app as an intervention tool should be considered as a complement, rather than a substitute of other means of support.

### Strengths and limitations

To achieve credibility in the analyses, all the co-authors in this study jointly discussed each of the steps of the analysis process. As possibly most of the participants who consented to participate in the study were digitally literate, the study may have excluded essential user perspectives on additional challenges of mobile apps as a mode of communication for providing and receiving support. Lastly, parts of the data collection for this study took place during the COVID-19 pandemic which was the main cause of the large number of telephone interviews. The telephone interviews were generally shorter than the video and face-to-face interviews which may have affected the richness of data of the former. However, recent research [47] suggests that telephone interviews provide the participants with relative anonymity that helps reduce inhibitions.

## Conclusions

The findings from this study can help community-based social care providers plan and organize long-distance support for FCs to PWD living at home. Furthermore, the findings support the use of a mobile app as a complement to traditional means of support for FCs to PWD which can facilitate their knowledge, awareness, and self-care management. Given the usefulness of the app STAV as experienced by both social care professionals and FCs, its integration in the care system would be a reasonable next step. Lastly, the findings of our study can also help healthcare providers, app developers, researchers, and other stakeholders within the service network in the development of sustainable mobile app-based interventions for the increasingly digitally literate FCs to PWD living at home.

### Electronic supplementary material

Below is the link to the electronic supplementary material.

Supplementary Material 1

## Data Availability

The datasets generated during/or analyzed during the current study are available from ZNK upon reasonable request.

## Abbreviations

App(s): Application(s)
FC(s): Family caregiver(s)
PWD: Person(s) with dementia
STAV: STöd till AnhörigVårdare: Support to family caregivers
