# Supplementary material for: Providing and receiving support through a tailor-made mobile app: a qualitative study on experience of professionals and family caregivers to persons with dementia
Source: BMC Geriatr. 2024 Jun 25;24:554. doi: 10.1186/s12877-024-05151-6 (PMC11202250; doi:10.1186/s12877-024-05151-6)
Supplement: Supplementary file 1 — Supplementary Material 1 [file 12877_2024_5151_MOESM1_ESM.docx]

**Supplementary Table 1** Interview guides for the family caregivers to persons with dementia and the social care professionals.

| Participant | Questions |
| --- | --- |
| Family caregivers to persons with dementia | - Can you describe your thoughts on the mobile app you have used? - Can you describe your thoughts on the content and features of the mobile app you have used? - How easy was it to use the app? Was it easy to understand? Was there anything that was difficult? - Can you describe whether the app has helped you, and in what way has it helped you? - Have you experienced that the support from the social care professional through the app has led to any changes in your well-being? - How do you experience the chat feature where you write your messages? - Can you describe how the communication between you and the social care professional worked? Can you describe a situation when it worked well, and a situation when it didn't work so well? - Did you experience any difference in the collaboration/relationship with the social care professional when you used the app? - Can you describe your experience of the diary feature? - Can you describe how you experienced the links in the app, what was good, and was there anything that could be improved? - Can you describe how you experienced the mindfulness feature? - Is there anything you missed in the app that you wish had been included? - Do you have suggestions for improving the app's content and usage? - Do you feel/assess that you have continued use of the app? - Would you recommend the app to a family caregiver in a similar situation to you? - Finally, is there anything else that you would like to elaborate on or something we have not mentioned? |
| Social care professionals | - Can you describe your experience of using the STAV app? - What expectations did you have before starting to use the app in your work? How did it turn out? - Could the app be useful in your work in the future? - What has been the biggest challenge in using the app? - What was the difficult part? - What was good about the app? - Looking back, can you describe how you experienced the contact with the family caregivers? - How was the support you provided through the app scheduled? - Were you the one who took the initiative to conduct the chat conversations? - How did you experience providing support to family caregivers through the chat? - Can you describe a situation when it worked well? What about that situation, do you think made the contact good? - Can you describe a situation when you felt that the contact with the family caregivers did not work very well? - What kind of contact did you have with family caregivers before the support through the STAV app? - When you communicated during the weeks of the project, did you communicate in any other way than through the app? - Can you describe any difference between the questions and messages you received through the STAV chat feature in comparison to what you usually receive over the phone and in physical meetings? - What do you think of the different app features? - Did the app affect your work situation in any way? - Is there anything in your contact with the family caregivers that you would have liked to have been different? - Is there anything in the app you would like to change or is there something you would like to add? - Finally, is there anything else that you would like to elaborate on that we have not talked about? |
